# Novel peptide dermaseptin‐PS1 exhibits anticancer activity via induction of intrinsic apoptosis signalling

**DOI:** 10.1111/jcmm.14032

**Published:** 2018-11-20

**Authors:** Qilin Long, Lei Li, Hao Wang, Miaoran Li, Lei Wang, Mei Zhou, Qiaozhu Su, Tianbao Chen, Yuxin Wu

**Affiliations:** ^1^ Natural Drug Discovery Group School of Pharmacy Queen's University Belfast Belfast UK; ^2^ Department of Nutrition and Metabolic Disease The Institute for Global Food Security School of Biological Sciences Queen's University Belfast Belfast UK

**Keywords:** antimicrobial peptides, apoptosis, concentration, dermaseptin, mitochondria

## Abstract

Antimicrobial peptides (AMP) secreted by the granular glands of frog skin have been widely reported to exhibit strong bacteriostatic and bactericidal activities. Many of them have been documented with potent antiproliferative effects on multiple cancer cells, many studies also suggested that AMPs exert their functions via disrupting cell membranes. However, whether and how other cell death induction mechanism is involved in mammalian cancer cells has rarely been investigated. In this study, a novel AMP named Dermaseptin‐PS1 was isolated and identified from *Phyllomedusa sauvagei*, it showed strong antimicrobial activities against three types of microorganisms. In vitro antiproliferative studies on human glioblastoma U‐251 MG cells indicated that Dermaseptin‐PS1 disrupted cell membranes at the concentrations of 10^−5^ M and above, while the cell membrane integrity was not affected when concentrations were decreased to 10^−6^ M or lower. Further examinations revealed that, at the relatively low concentration (10^−6^ M), Dermaseptin‐PS1 induced apoptosis through mitochondrial‐related signal pathway in U‐251 MG cells. Thus, for the first time, we report a novel frog skin derived AMP with anticancer property by distinct mechanisms, which largely depends on its concentration. Together, our study provides new insights into the mechanism‐illustrated drug design and the optimisation of dose control for cancer treatment in clinic.

## 1 INTRODUCTION

Glioblastoma, as the major type of primary brain tumour, has the worst prognosis among all malignancies. However, due to the abnormal cellular composition and rapid escape from therapeutic agents, very limited progress has been made in the last few decades for effectively killing or controlling glioblastoma cells. To date, combining the radiotherapy and chemotherapy with surgery remains the mainstream strategy for the treatment of glioblastoma.^1^, ^2^ Therefore, development of novel drugs for specifically targeting human glioblastoma has promising perspective.

The de novo design of AMPs from natural sources has brought much attention, as many of these peptides share the properties of being linear, cationic and α‐helical, which are essential for the initial attraction of them to the negatively charged cell membrane for consequent membrane disruption.^3^ It is well established that prokaryotic cell membrane and eukaryotic mitochondrial membrane possess high transmembrane potentials. By contrast, the zwitterionic phospholipid, which is the main composition of eukaryotic membrane, possesses low transmembrane potential.^4^, ^5^, ^6^, ^7^ As a consequence, the majority of AMPs prefer to corrupt the prokaryotic membrane and eukaryotic mitochondrial membrane rather than the eukaryotic membrane. Thus, those AMPs with distinct membrane‐penetration mechanism on tumour cells that allows the receptor‐regulated internalisation, followed by induction of programmed cell death, could serve as unique and supplementary options for cancer therapy.

Apoptosis is a programmed process that plays a pivotal role in modulating physiological and pathological cell death.^8^ Extensive scrutiny has been focused on the investigation of apoptosis‐related cancer research, in particular the disorder of cancer cells to undergo apoptosis could lead to severe malignant progression and chemotherapy resistance.^9^ The whole process of apoptosis composed of either of the two distinguished signalling transduction pathways and a number of proteins that located in multiple organelles.^10^ Compared with death receptor (extrinsic) pathway, the mitochondrial (intrinsic) pathway of apoptosis is most commonly deregulated in cancer cells, and most stimuli induce the initiation of apoptosis through mitochondrial signalling that governs the release of cytochrome c to the cytosol, which in response regulates the activity of caspase protease.^11^, ^12^ Upon activation, caspases cleave a number of proteins, resulting in cell death, and the alterations in biochemistry and morphology occur.^13^

In this study, using human glioblastoma U‐251 MG cells, we investigated the tumour suppression effect of the novel isolated AMP Dermaseptin‐PS1 from *P. sauvagei*. We provide the evidence that the concentration is critical for anticancer agents to exert biological functions through differentiated mechanisms, which support the further drug design and development for targeting the intervention of mitochondrial apoptosis in cancer therapy.

## 2 MATERIALS AND METHODS

### 2.1 Specimen biodata and skin secretion harvesting

Specimens of *P. sauvagei* (n = 3, two males 5 cm snout‐to‐vent length, one female 7 cm snout‐to‐vent length), originally from South America, were obtained from a commercial source in the United States. The skin secretion produced by the holocrine glands of frogs was obtained by mild transdermal electrical stimulation (5 V, 50 Hz, 4 ms plus width) as previously described.^14^ Secretions were then collected by rinsing with distilled deionized water and were subjected to snap frozen with liquid nitrogen, lyophilized and stored at −20°C prior to use. Sampling of skin secretion was performed by Mei Zhou under UK Animal (Scientific Procedures) Act 1986, project license PPL 2694, issued by the Department of Health, Social Services and Public Safety, Northern Ireland. Procedures had been vetted by the IACUC of Queen's University Belfast and approved on 1 March 2011.

### 2.2 “Shotgun” cloning of novel Dermaseptin‐like peptide encoding cDNAs from lyophilized skin secretion

Five milligrams of lyophilised *P. sauvagei* secretion powder were dissolved in 1 mL cell Lysis/Binding buffer to isolate polyadenylated mRNA by using magnetic oligo‐dT beads in Dynabeads^®^ mRNA DIRECT™ Kit (Dynal Biotech, Liverpool, UK). Then the reverse‐transcribed cDNA library was subjected to 3′‐RACE PCR procedures to acquire the full length of preproprotein nucleic acid sequences using a SMART‐RACE kit (Clontech, Palo Alto, CA, USA) essentially as described by the manufacturer. Briefly, the 3′‐RACE reactions employed a UPM primer (supplied with the kit) and degenerate sense primers (S1; 5′‐ACTTTCYGAWTTRYAAGMCCAAABATG‐3′, Y = C + T, W = A + T, R = A + G, M = A + C, B = T + C + G) that was designed to a segment of the 5′‐untranslated region of phylloxin cDNA from *Phyllomedusa bicolor* (EMBL accession no. AJ251876) and the opioid peptide cDNA from *Pachymedusa dacnicolor* (EMBL accession no. AJ005443).^15^ PCR cycling procedures were carried out as follows: initial denaturation step: 90 seconds at 94°C; 35 cycles: denaturation 30 seconds at 94°C, primer annealing for 30 seconds at 58°C; extension for 180 seconds at 72°C. PCR products were gel‐purified and cloned using a pGEM‐T vector system (Promega Corporation, Southampton, UK), and selected samples were sequenced by an ABI 3730 automated sequencer.

### 2.3 Identification and structural characterisation of the novel Dermaseptin‐like peptide

An aliquot sample of the lyophilised *P. sauvagei* skin secretion was dissolved in 1 mL of trifluoroacetic acid (TFA)/water (0.05:99.95, v/v) and clarified by centrifugation. One millilitre of clear supernatant was carefully decanted and pumped directly into a reverse‐phase HPLC column (C‐18, 300 Å, 5 μm, 4.6 mm × 250 mm; Phenomenex, Cheshire, UK). The elution gradient formed from 0.05/99.5 (v/v) TFA/water to 0.05/19.95/80.0 (v/v/v) TFA/water/acetonitrile in 240 minutes at a flow rate of 1 mL/min and the effluent was detected by UV absorbance at 214 nm and 280 nm. An automatic fraction collector (GE Healthcare, Little Chalfont, UK) was used to collect the fractions at 1‐minute interval. All fractions were interrogated by matrix‐assisted laser desorption ionisation time‐of‐flight (MALDI‐TOF) mass spectrometry in positive detection mode using alpha‐cyano‐4‐hydroxycinnamic acid (CHCA) as matrix. The fractions with masses coincident with the putative peptide from molecular cloning were subjected to Liquid Chromatography Quadruple (LCQ)‐Fleet electrospray ion‐trap mass spectrometer (Thermo Fisher Scientific, San Francisco, CA, USA) for primary structural analysis.

### 2.4 Solid‐phase peptide synthesis

Following unambiguous confirmation of the primary structure through both molecular cloning strategy and LCQ‐Fleet mass spectrometry, the peptide was chemically synthesised by Tribute™ automated solid phase peptide synthesizer 4 (Protein Technologies, Tucson, AZ, USA). The synthesised peptide replicates were then purified by reverse‐phase HPLC and confirmed by MALDI–TOF mass spectrometry prior to use.

### 2.5 Peptide secondary structure determination via circular dichroism

JASCO J‐815 circular dichroism (CD) spectrometer (Jasco, Essex, UK) was used to detect the secondary structure of Dermaseptin‐PS1. Peptide was dissolved in (a) 10 mmol/L NH_4_AC, (b) 50% (v/v) trifluoroethanol (TFE)‐10 mmol/L NH_4_AC to reach a final concentration of 100 μmol/L before transferred and measured in a 0.1 cm high precision quartz cell (Hellma Analytics, Essex, UK). The wavelengths used were from 190 nm to 260 nm with a scanning speed of 200 nm/min, and the bandwidth and data pith were 1 nm and 0.5 nm respectively. CD data are expressed as the molar ellipticity [θ] in deg/cm^2^/dmol at respective wavelength (nm), which is calculated from the measured Ellipticity (θ, in medg) using the equation [θ] = θ/(10 × *c* × *l*) where *c* is the molar concentration of the sample (mol/L) and *l* is the cuvette path length in centimetre (cm). DICHROWEB webserver (http://dichroweb.cryst.bbk.ac.uk/html/home.shtml) was used to estimate the contents of different secondary structures.^16^, ^17^, ^18^

### 2.6 Antimicrobial and haemolytic assays

The antimicrobial assays for evaluating the minimum inhibitory concentration (MIC) and the minimum bactericidal concentration (MBC) of the peptide were performed by using the Gram‐positive bacterium *Staphylococcus aureus* (*S. aureus*) (NCTC 10788), the Gram‐negative bacterium, *Escherichia coli* (*E. coli*) (NCTC 10418) and the yeast, *Candida albicans* (*C. albicans*) (NCPF 1467). The haemolytic activity of Dermaseptin‐PS1 was assessed using a 4% suspension of horse red blood cells (supplied by TCS Biosciences Ltd, Botolph Claydon, Buckingham, UK) in phosphate‐buffered saline (PBS). The detailed procedures were described previously.^19^

### 2.7 Cell line, cell culture and chemicals

Human glioblastoma cells, U‐251 MG, were purchased from the cell bank of European Collection of Authenticated Cell Cultures (ECACC‐09063001) in 2015. The cells were authenticated by ECACC cell bank using short tandem repeat polymorphism analysis and were expanded and stored in liquid nitrogen upon receipt, and each aliquot was passaged for fewer than 6 months in our laboratory. Once resuscitated, the cell line was authenticated by monitoring the cell morphology. U‐251 MG was cultured in Dulbecco's Modified Eagle's Medium (DMEM) (Invitrogen, Paisley, UK) containing 2 mmol/L Glutamine (gibco^®^, Paisley, UK), 1% non‐essential amino acids (NEAA) (gibco^®^), 1 mmol/L sodium pyruvate (NaP) (Thermo Fisher Scientific), 10% foetal bovine serum (FBS) (gibco^®^) and 1% antibiotics (100 U/mL penicillin, 0.1 mg/mL streptomycin; gibco^®^) at 37°C with 5% CO_2_. Etoposide was purchased from Tocris Bioscience, Bristol, UK (Cat: 1226), and Z‐VAD‐FMK was purchased by Dr Mei Chen in WWIEM, Queen's University Belfast from InvivoGen, Toulouse, France (Cat: tlrl‐vad).

### 2.8 MTT cell proliferation and LDH cell membrane integrity evaluations

Five thousand cells/well in 100 μL full growth DMEM medium were planted into 96‐well plates and allowed to attach for 24 hours. Cells were starved by serum‐free medium for 6 hours before treated with peptide concentration gradient (10^−4^ to 10^−9^ M, n = 5) for 24 hours. After which, 10 μL of 3‐(4,5‐dimethylthiazol‐2‐yl)‐2,5‐diphenyltetrazolium bromide (MTT) solution was added to each well prior to another 4 hours incubation, the optical density (OD) values were measured at 570 nm by using EL808 TM Absorbance Microplate Reader.

The cell membrane integrity was measured by using lactate dehydrogenase (LDH) assay with Pierce LDH Cytotoxicity Assay Kit (Thermo Scientific, Loughborough, UK) according to the manufacturer's instruction with the same treatment conditions.

### 2.9 Western blotting

Protein lysates extracted from cells were used to detect protein expression levels of caspase 3 (Cell Signaling, Stillorgan, Ireland; #9662), caspase 8 (Cell Signalling; #9746), FADD (Cell Signalling; #2782), caspase 9 (Cell Signalling; #9508), Apaf‐1 (Cell Signalling; #8723), Bcl‐2 (Cell Signalling; #4223), Bax (Cell Signalling; #5023), Bak (Cell Signalling; #3814), Bad (Cell Signalling; #9292), Phospho‐Bad (Ser112) (Cell Signalling; #9291), Phospho‐p53 (Ser15) (Cell Signalling; #9284), p53 (Cell Signalling; #2524), cytochrome c (Cell Signalling; #4272), COX IV (Cell Signalling; #4844), β‐Tubulin (Cell Signalling; #2146) and GAPDH (Cell Signalling; #5174). The immunoblotting protocol was previously described.^19^

### 2.10 Annexin V apoptosis fluorescence imaging

Cells were planted into 4‐well chamber‐slides (Falcon^®^ Culture Slides, VWR, UK) at a density of 6 × 10^4^ cells/mL/well and allowed to attach for 24 hours. After 6 hours starvation, cells were treated with peptide concentration gradient (10^−4^ to 10^−7^ M) for 16 hours. Then, cells were dyed with FITC‐Annexin V Apoptosis Detection Kit I (BD Pharmingen™; BD Biosciences, Berkshire, UK) according to the manufacturer's instruction. After the staining, 4% paraformaldehyde (Alfa Aesar, Ward Hill, MA, USA) was used to fix cells and one drop of anti‐fade mounting medium (Thermo Fisher, UK) was added to each well before coverslip seal using nail polish. The slides were immediately analysed by Leica DMi8 fluorescent microscope imaging system.

### 2.11 RNA extraction and quantitative real‐time PCR analysis

Cells were planted into 6‐well plates at a density of 2 × 10^5^ cells/2 mL/well and allowed to attach for 24 hours. After 6 hours starvation, cells were treated by peptide concentration gradient (10^−4^ to 10^−7^ M) for 16 hours. Then, 500 μL of TRIzol^®^ Reagent (Thermo Fisher, Loughborough, UK) was added into each well and RNA was extracted by 100 μL chloroform and purified by 200 μL isopropanol. The solutions were centrifuged at 12 000 × *g* for 10 minutes and the supernatant was removed before the pellets were washed with 75% ethanol. After which, the RNA pellets were resuspended in 20 μL diethyl pyrocarbonate (DEPC) water and quantified using Nanodrop One^c^ system (Thermofisher, UK). One microgram RNA from each sample was reverse transcribed by using iScript™ cDNA Synthesis Kit (Bio‐Rad, Dublin, Ireland) according to the manufacturer's instructions. Real‐time PCR amplification was performed with SYBR^®^ Select Master Mix (Thermofisher) on an CFX Connect™ Real‐Time PCR Detection System (Bio‐Rad) with PCR procedures under the following programme: 1 cycle at 95°C for 3 minutes; 45 cycles at 95°C, 20 seconds and 60°C for 1 minute; 1 cycle at 95°C for 1 minute and 55°C for 1 minute; 80 cycles at 55°C for 10 seconds and increase set point temperature after cycle 2 by 0.5°C. The melt curve data were collected and analysed. The relative mRNA abundance normalized to 18S rRNA levels was determined with the ΔΔCt (cycle threshold) method after amplification. Data are represented as mean ± SD. Sequences for primers used in this study are listed in Table 1.

**Table 1:** The primer sequences used in conducting the qPCR experiments

Target protein	Sense primer	Antisense primer
APAF‐1	5′‐CCTGTTGTCTCTTCTTCCAGTGT‐3′	5′‐AAAACAACTGGCCTCTGTGG‐3′
TNFR1	5′‐TGCCAGGAGAAACAGAACAC‐3′	5′‐TCCTCAGTGCCCTTAACATTC‐3′
Fas	5′‐ACTCACCAGCAACACCAAG‐3′	5′‐TCATGACTCCAGCAATAGTGG‐3′
Bax	5′‐GAGCAGATCATGAAGACAGGG‐3′	5′‐AGTAGAAAAGGGCGACAACC‐3′
Bcl‐2	5′‐GTGGATGACTGAGTACCTGAAC‐3′	5′‐CCTGCAGCTTTGTTTCATGG‐3′
Bid	5′‐ATTAACCAGAACCTACGCACC‐3′	5′‐TCTAGGAACGCTGTTGACATG‐3′
Caspase 8	5′‐ATCCTGAAAAGAGTCTGTGCC‐3′	5′‐ATTCCTGTCCCTAATGCTGTG‐3′
Caspase 9	5′‐CCTAGAAAACCTTACCCCAGTG‐3′	5′‐CACGGCAGAAGTTCACATTG‐3′
18S	5′‐CGGCTACCACATCCAAGGAA‐3′	5′‐AGCTGGAATTACCGCGGC‐3′

### 2.12 Cytosolic and mitochondrial protein extraction

About 5 × 10^6^ cells were treated with 500 μL Mitochondrial Protein Isolation Buffer (2 μL 100× protease inhibitors added) (AMRESCO, Lutterworth, Leicestershire, UK) and incubated on ice for 10 min. The cell membrane was lysed by sucking the solution back and forth for 20 times with a 26 ½ G needle. The cell debris including the unbroken nuclei was removed through centrifugation at 1000 × *g*, 4°C for 10 minutes. The cytoplasmic proteins were collected from the supernatant after the solution was further centrifuged at 15 000 × *g*, 4°C for 20 minutes. The mitochondrial proteins were collected from the pellet treated with 100 μL of Mitochondrial Protein Isolation Buffer.

### 2.13 Statistical analysis

All results are presented as mean ± SEM determined by two‐tailed Student's *t* tests or one‐way anova. Pair comparisons of the means were made, and *P* < 0.05 was taken as a significant difference. The Bonferroni method was used to adjust the observed significance levels for the fact the multiple contrasts were being tested.

## 3 RESULTS

### 3.1 Identification, characterisation and chemical synthesis of the novel peptide Dermaseptin‐PS1 from *P. sauvagei*

**Figure 1:**
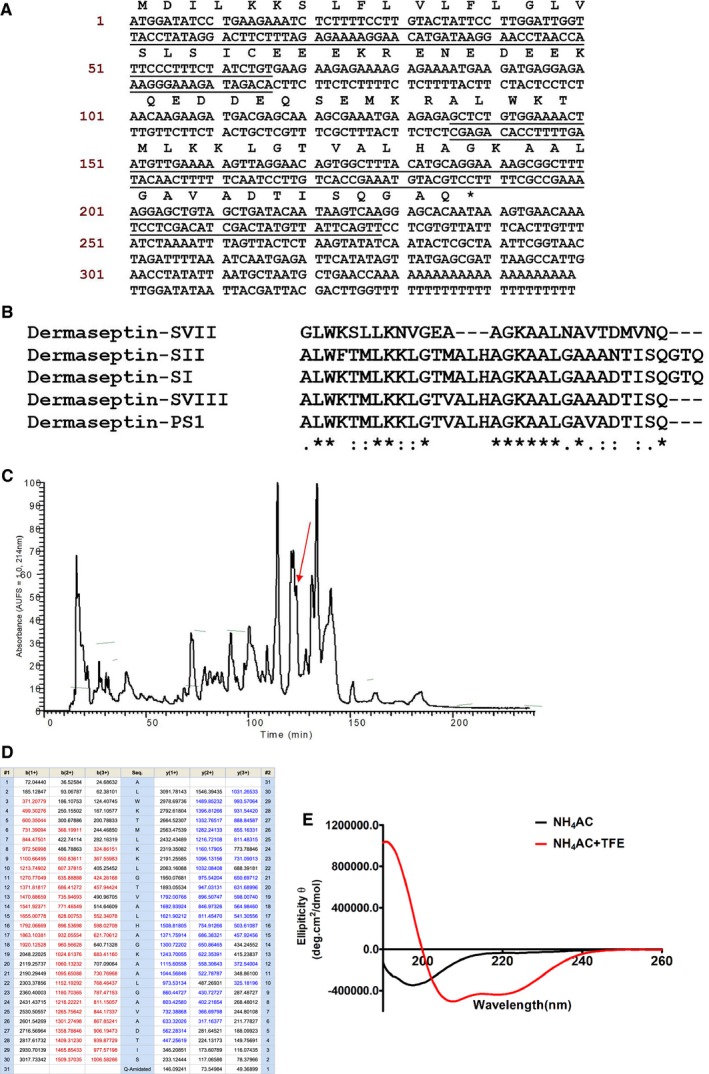
Primary structure identification, determination and analysis of Dermaseptin‐PS1 from *Phyllomedusa sauvagei* (A) nucleotide and translated open‐reading frame amino acid sequences of cloned cDNA encoding the Dermaseptin‐PS1 precursor from *P. sauvagei* skin secretion (EMBL accession number: LT840240). The putative signal peptide is double‐underlined, the mature peptide is single‐underlined and the stop codon is indicated by an asterisk. (B) Alignment of cDNA deduced mature Dermaseptin‐PS1 sequence with dermaseptin peptides from *P. sauvagei* species. The identical and conservative residues are indicated by asterisks (*) and full stop (.)/colon (:), respectively. (C) rp‐HPLC chromatogram of *P. sauvagei* skin secretion with an arrow indicating the retention time (at 123 min) of Dermaseptin‐PS1. The detection wavelength was 214 nm with a flow rate of 1 mL/min in 240 min. (D) Predicted single‐ and double‐charged *b*‐ and *y*‐ion series arising from LCQ MS/MS fragmentation of Dermaseptin‐PS1. The truly observed fragment ions following actual fragmentation are shown in red (*b*‐ions) and blue (*y*‐ions) typefaces respectively. (E) The CD spectra recorded for the purified synthetic Dermaseptin‐PS1 in (a) 10 mmol/L ammonium acetate (NH_4_Ac) water solution and (b) 50% 2,2,2‐trifluoroethanol (TFE)‐50% NH_4_Ac water solution. The molar ellipticity was plotted against wavelength and the tested peptide concentration was 100 μmol/L

The cDNA library was constructed using the skin secretion of *P. sauvagei*, from which, the cDNA encoding the biosynthetic precursor of Dermaseptin‐PS1 was consistently cloned (n = 6). The coding region of the cDNA composed of 79 amino acids. It consists of four domains: a 19‐residue putative signal peptide domain located at the N‐terminus of the reading frame, a 23‐residue acidic spacer domain which is followed by the canonical propeptide convertase processing site Lys‐Arg (K‐R), a 31‐mer mature peptide domain with glycine‐76 acts as an amide donor, and a C‐terminal untranslated region. The predicted mature peptide sequence was determined as: ALWKTMLKKLGTVALHAGKAALGAVADTISQ‐NH_2_ (Figure 1A). Via BLAST and Clustal Omega sequence alignment analysis, the sequence of the mature peptide showed high similarity with Dermaseptin‐SVII (EMBL accession number: AJ564792), Dermaseptin‐SII (P80278), Dermaseptin‐SI (P80277) and Dermaseptin‐SVIII (AJ564792) from *P. sauvagei*, which suggested that this novel peptide belongs to the Dermaseptin family, and we named it as Dermaseptin‐PS1 (Figure 1B).

The fractionated skin secretion of *P. sauvagei* by reverse‐phase (rp) HPLC (Figure 1C) was subjected to MALDI‐TOF mass spectrometry analysis, the fractions that share the identical molecular weight with the deduced mature peptide from cloning were further examined by LCQ‐Fleet electrospray ion‐trap mass spectrometer for primary structure identification, as Figure 1D shown, the mature sequence of Dermaseptin‐PS1 was unequivocally confirmed with the C‐terminal amidation after analysis of multiply charged pre‐cursor fragmentation spectra using Max‐Ent3 software (Waters). The nucleotide sequence of the encoding cDNA precursor of Dermaseptin‐PS1 from the skin secretion of *P. sauvagei*, has been deposited to EMBL Nucleotide Sequence Database under the accession code: LT840240.

For further biological function investigations, the Dermaseptin‐PS1 peptide replicates were chemically synthesised by solid‐phase Fmoc strategy through Tribute™ automated solid‐phase peptide synthesizer 4 (Protein Technologies). The crude synthetic products were purified and confirmed by rp‐HPLC and MALDI‐TOF MS (Figure S1), respectively, to achieve the purity >95%.

### 3.2 Secondary structure determination using CD analysis

Circular dichroism was used to investigate the secondary structures of Dermaseptin‐PS1 in 10 mmol/L ammonium acetate (NH_4_Ac) (mimicking aqueous environment) and 50% TFE‐10 mmol/L NH_4_Ac solution (mimicking the hydrophobic environment of cell membrane) solutions (Figure 1E). The generated data were analysed by DICROWEB webserver. In 10 mmol/L NH_4_Ac solution, Dermaseptin‐PS1 showed a mixed conformation of random coil (48%) and β‐sheet (48%) with a negative band presented at 198 nm. By contrast, in 50% TFE‐10 mmol/L NH_4_Ac solution, the α‐helical structure was increased to 25%, and random coil increased to 60%, while β‐sheet decreased to 15% (Table 2). The results suggested that the Dermaseptin‐PS1 was partially structured to a membrane‐penetrated β‐sheet structure in membranes mimic surroundings, but largely remained unstructured in aqueous solution, indicating that Dermaseptin‐PS1 did not adopt a typical antimicrobial structure (α‐helix or β‐sheet) in physiological condition, and therefore, acting as a ligand for receptor activation might be the main underlying mechanism of its bioactivity.

**Table 2:** Secondary structure analysis of Dermaseptin‐PS1 by using DICHROWEB online server

Percentage (%)	α‐helix	β‐sheet	Random coil
NH_4_Ac	4	48	48
50% TFE +NH_4_Ac	25	15	60

### 3.3 Dermaseptin‐PS1 exhibited moderate antimicrobial activity against typical strains

**Figure 2:**
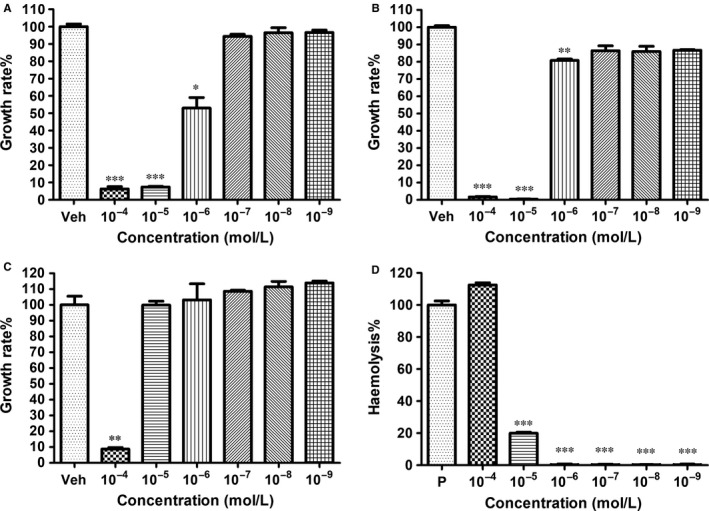
Antimicrobial activity and haemolytic effect of Dermaseptin‐PS1. The minimum inhibitory concentrations of Dermaseptin‐PS1 against (A) *Staphylococcus aureus*, (B) *Escherichia coli* and (C) *Candida albicans*. The minimum bactericidal concentration (MBC) values against *S. aureus*,* E. coli* were 10^−4^ M and no MBC value was detected against *C. albicans*. Data were analysed with unpaired Student's *t* test using GraphPad Prism 5 software. Values are the mean ± SEM for three independent experiments. Veh represents vehicle control, *P < 0.05, **P < 0.01 and ***P < 0.001 vs vehicle control. (D) Haemolytic activity of Dermaseptin‐PS1 following incubation with horse erythrocytes for 2 h. Data were analysed with unpaired Student's *t* test using GraphPad Prism 5 software. Values are the mean ± SEM for three independent experiments. P represents positive control, ***P < 0.001 vs positive control (10% Triton X‐100)

As Dermaseptin family peptides have been widely reported to possess strong antimicrobial activities,^20^, ^21^, ^22^ we tested whether Dermaseptin‐PS1 can retard the reproduction of microorganisms. The results showed that the novel peptide Dermaseptin‐PS1 exhibited moderate antimicrobial activities against *S. aureus* (Figure 2A), *E. coli* (Figure 2B) and *C. albicans* (Figure 2C) with relatively low haemolytic effect (<20% at 10^−5^ M) (Figure 2D). The MIC values for the Gram‐positive (*S. aureus*) and the Gram‐negative bacteria (*E. coli*) were 10^−5^ M, which is higher than the 10^−4^ M against the yeast (*C. albicans*), and the MBC values of Dermaseptin‐PS1 against *S. aureus*,* E. coli* were 10^−4^ M, no MBC value was detected against *C. albicans*.

### 3.4 Dermaseptin‐PS1 displayed antiproliferative activity in glioblastoma U‐251 MG cells

**Figure 3:**
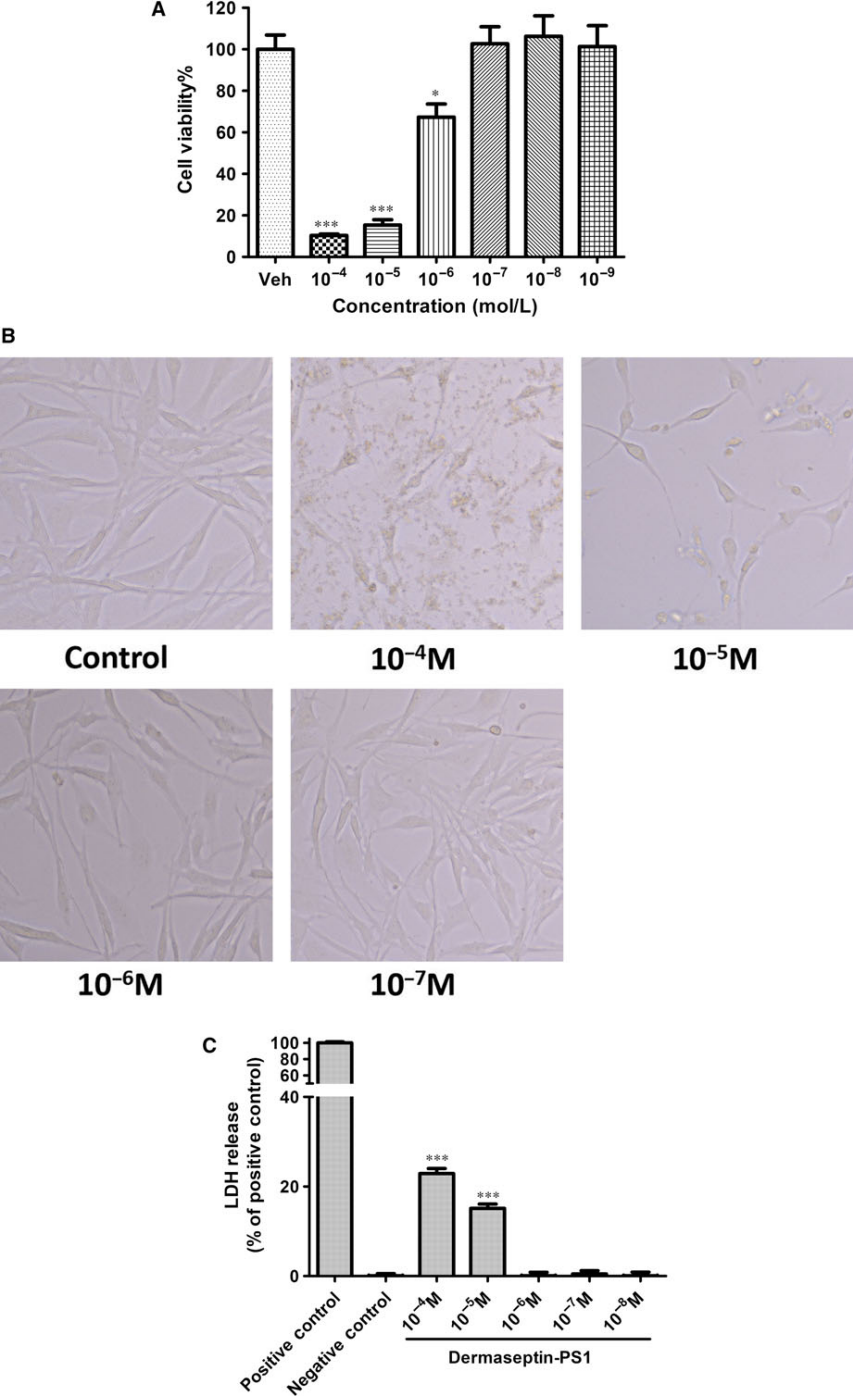
Dermaseptin‐PS1 display antiproliferative activity against U‐251 MG cell line. A, The antiproliferative effect of human neuronal glioblastoma cells, U‐251 MG cell line after the treatment of Dermaseptin‐PS1 gradient (10^−4^ to 10^−9^ M) for 24 h. The calculated IC_50_ value was 5.419 μmol/L. Data were analysed with unpaired Student's *t* test using GraphPad Prism 5 software. Values are the mean ± SEM for three independent experiments. Veh represents vehicle control, ***P < 0.001 vs vehicle control. B, The morphology changes of U‐251 MG cells after the treatment of Dermaseptin‐PS1 gradient (10^−4^ to 10^−7^ M) for 24 h. The images were observed by a magnification factor ×200. C, Lactate dehydrogenase (LDH) release from U‐251 MG cells after the treatment of Dermaseptin‐PS1 gradient (10^−4^ to 10^−8^ M) for 24 h. Data were analysed with unpaired Student's *t* test using GraphPad Prism 5 software. Values are the mean ± SEM for three independent experiments. ***P < 0.001 vs negative control

Many of the AMPs, with the inherent characteristics of being linear, cationic and amphipathic, have been proved to exert antiproliferative capacities through non‐specific interaction with eukaryotic cell membranes,^23^ while internalisation initiated apoptosis induction has also been reported to act as the major mechanism of anticancer functions.^24^ Thus, we tested the antiproliferative activity of Dermaseptin‐PS1 using different cell lines, and the results (Figure 3 and Figure S2) indicated that the glioblastoma U‐251 MG cells have lowest survival rates after Dermaseptin‐PS1 treatment. MTT cell viability examinations revealed that Dermaseptin‐PS1 showed strong antiproliferative effects on all tested cancer cell lines and human mammary epithelial cell (HMEC) at 10^−4^ M and 10^−5^ M concentrations, however, at the concentration of 10^−6^ M, a moderate antiproliferation activity was observed in U‐251 MG cells (cell viability around 65%) after Dermaseptin‐PS1 treatment (Figure 3A), while the cell viabilities of other cancer cells and HMEC cells were not affected (Figure S2), which reminded us that Dermaseptin‐PS1 might have specific cell death induction mechanism in U‐251 MG cancer cells at 10^−6^ M. In addition, when the tested peptide concentration decreased to 10^−7^ M or lower, the antiproliferative effect of Dermaseptin‐PS1 on all tested cell lines was completely abrogated. To further explore whether the cell viability reduction was caused by the cancer cell membrane disruption, we employed phase contrast microscopy and lactate dehydrogenase (LDH) assay to assess the cell membrane integrity in U‐251 MG cells. As shown in Figure 3B, after 24 hours treatment of 10^−4^ and 10^−5^ M Dermaseptin‐PS1, the U‐251 MG cells distributed sparsely, lost cell‐cell contact, became rounded, and much more cellular debris was observed compared with control group and 10^−6^ M, 10^−7^ M groups, which appeared to retain an integral cell shape. This suggested that the cell membranes ruptured by high concentrations of Dermaseptin‐PS1, while when the concentration decreased to 10^−6^ M and lower, the morphological integrity of the cells was not affected. Moreover, as has been documented that one of the key signatures of cells undergoing necrosis is the cell membrane permeabilisation, which could be simply detected through LDH release assay.^25^ Therefore, we performed LDH assay to assess whether at high concentration (10^−5^ M) Dermaseptin‐PS1 kills cells via necrosis. The results (Figure 3C) showed that, at the concentration of 10^−4^ M, Dermaseptin‐PS1 caused more than 20% LDH release from the cells, and 10^−5^ M concentration also triggered a more than 15% leakage. In contrast, 10^−6^ to 10^−8^ M did not render LDH release, indicating a obvious necrosis induction mechanism by Dermaseptin‐PS1 at the concentration of 10^−5^ M and above. Therefore, these results prompt us to suggest that Dermaseptin‐PS1 may have a distinct U‐251 MG cells killing mechanism, which is mainly in a concentration‐related manner.

### 3.5 Dermaseptin‐PS1 induced U‐251 MG cell death at 10^−6^ M via induction of apoptosis

**Figure 4:**
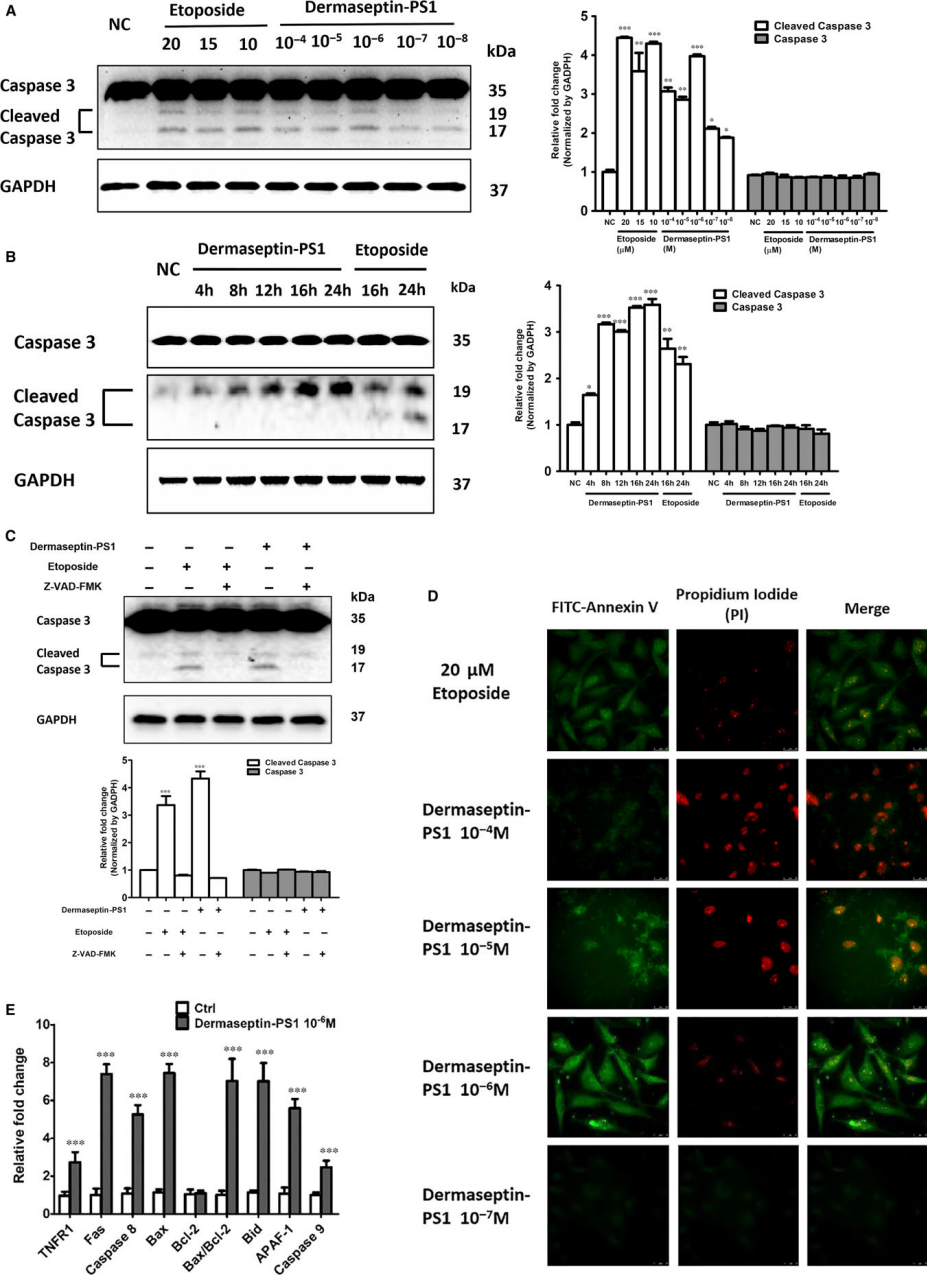
Dermaseptin‐PS1 induced U‐251 MG cell death at 10^−6^ M via apoptosis. Western blot analysis was performed on U‐251 MG cells to test the expression of cleaved caspase 3 and caspase 3 after the treatment of (A) NC, 20 μmol/L, 15 μmol/L and 10 μmol/L etoposide and 10^−4^ to 10^−8^ M Dermaseptin‐PS1 for 24 h; (B) 10^−6^ M Dermaseptin‐PS1 and 20 μmol/L etoposide for indicated times; (C) 20 μmol/L Z‐VAD‐FMK for 2 h subsequent to 20 μmol/L etoposide and 10^−6^ M Dermaseptin‐PS1 treatment for 16 h. The detection of GAPDH protein was used as an internal control. The signal intensity was quantified by Image Lab software and GraphPad Prism 5 software was used for statistical comparison. NC, negative control. *P < 0.05, **P < 0.01 and ***P < 0.001 vs negative control. (D) The fluorescent microscopy images showing FITC‐annexin V positive membranes, propidium iodide (PI)‐stained nuclei and a merge image. The images were captured from a population of U‐251 MG with the exposure to Dermaseptin‐PS1 gradient (10^−4^ to 10^−7^ M) or 20 μmol/L etoposide for 16 h. Scale bar = 25 μm. (E) The mRNA levels of apoptosis‐related genes were analysed by real‐time PCR. The qPCR analysis performed on U‐251 MG cells by the treatment of 10^−6^ M Dermaseptin‐PS1 for 16 h. The sequences of pro‐apoptotic genes are displayed in Table 1. The mRNA expression of 18S was used as a calibration standard. Data were analysed with one‐way anova using GraphPad Prism 5 software. Values are the mean ± SEM for three independent experiments. ***P < 0.001 vs negative control

Apoptosis and autophagy are two major programmed cell‐destructive processes, both of them could be elicited by exogenous stress or ligand.^26^ We firstly used Western blotting to examine whether Dermaseptin‐PS1 caused cell viability decrease was through the activation of either or both of apoptosis and autophagy. As the dose assessment shown in Figure 4A, Dermaseptin‐PS1 treatment at 10^−6^ M elevated the expression level of cleaved caspase 3. Quantitative analysis normalised by GAPDH revealed that, compared with negative control, 10^−8^ M, 10^−7^ M, 10^−5^ M and 10^−4^ M, the 10^−6^ M and etoposide (positive control) treatments have significant higher expression of the apoptosis marker cleaved caspase 3, and the total caspase 3 protein level was not affected. Additional time‐course assessment indicated that 10^−6^ M Dermaseptin‐PS1 induced the highest expression of cleaved caspase 3 after 16 hours and/or 24 hours treatment (Figure 4B). Based on these observations, we treated the U‐251MG cells using 10^−6^ M concentration of Dermaseptin‐PS1 for 16 hours with or without Z‐VAD‐FMK, a caspase inhibitor which could inhibit the induction of apoptosis.^27^ The results (Figure 4C) showed that, pre‐Z‐VAD‐FMK treatment completely abrogated Dermaseptin‐PS1 and etoposide‐induced elevation of cleaved caspase 3, suggesting the induction of apoptosis by Dermaseptin‐PS1 at 10^−6^ M in U251‐MG cells. However, autophagy activation assessment revealed that, compared with positive control (Brefeldin A or Earle's balanced salt solution), the LC3‐II/LC3‐I ratio did not increase after time‐course treatment of Dermaseptin‐PS1 on U‐251 MG cells (Figure S3). Thus, we suggested that Dermaseptin‐PS1 could activate apoptosis, rather than autophagy, to induce programmed cell death at the concentration of 10^−6^ M.

Furthermore, Annexin V‐based luminescent images showed that exposure of U‐251 MG cells at 10^−6^ M Dermaseptin‐PS1 or etoposide could elicit strong Annexin V signals (green), which were mostly localised on the membrane, while the propidium iodide (PI) was barely seen (red) in the nucleus. However, exposure of the cells to 10^−4^ M or 10^−5^ M Dermaseptin‐PS1 significantly diminished the Annexin V signals, by contrast, the nuclear PI staining signals were markedly elevated. While, 10^−7^ M Dermaseptin‐PS1 caused neither Annexin V nor PI signal increase (Figure 4D).

Next, we examined the related marker genes for both intrinsic and extrinsic cascades of apoptosis at transcriptional level. As shown in Figure 4E, the pro‐apoptotic genes were increased significantly, while the anti‐apoptotic Bcl‐2 gene remained unaltered, which lead to the elevation of Bax/Bcl‐2 ratio. The up‐regulation of Bax/Bcl‐2 ratio contributes to the activation of apoptotic cascade.^28^

Together, we confirmed that Dermaseptin‐PS1 disrupted the U‐251 MG cell membranes at 10^−5^ M and higher concentrations, and induced apoptosis at 10^−6^ M to exert the antiproliferative effects.

### 3.6 Dermaseptin‐PS1 induced U‐251 MG cell apoptosis through intrinsic cascade

**Figure 5:**
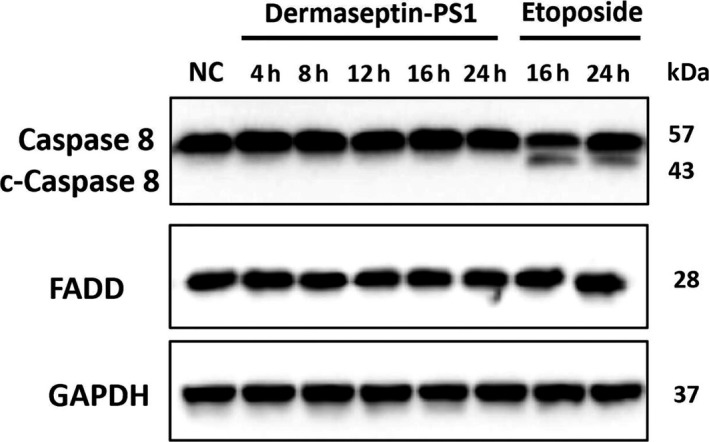
The examinations of extrinsic apoptotic cascade mediated by Dermaseptin‐PS1 in U‐251 MG cells. Protein expression of caspase 8/cleaved caspase 8 and FADD were analysed by Western blot in U‐251 MG cells treated for 4‐24 h with 10^−6^ M Dermaseptin‐PS1 or 16‐24 h with 20 μmol/L etoposide. The detection of GAPDH protein was used as an internal control

To investigate the specific intracellular mechanism by which Dermaseptin‐PS1 exerts the apoptosis induction effect, we examined the marker genes that related to the intrinsic or extrinsic apoptotic cascades. As shown in Figure 5, the extrinsic cascade related Caspase 8 and Fas‐associated protein with death domain (FADD) remained unchanged after 10^−6^ M Dermaseptin‐PS1 treatment for 16 hours. Thus, we suggested that the intrinsic signalling might be crucial for executing Dermaseptin‐PS1 triggered apoptosis.

**Figure 6:**
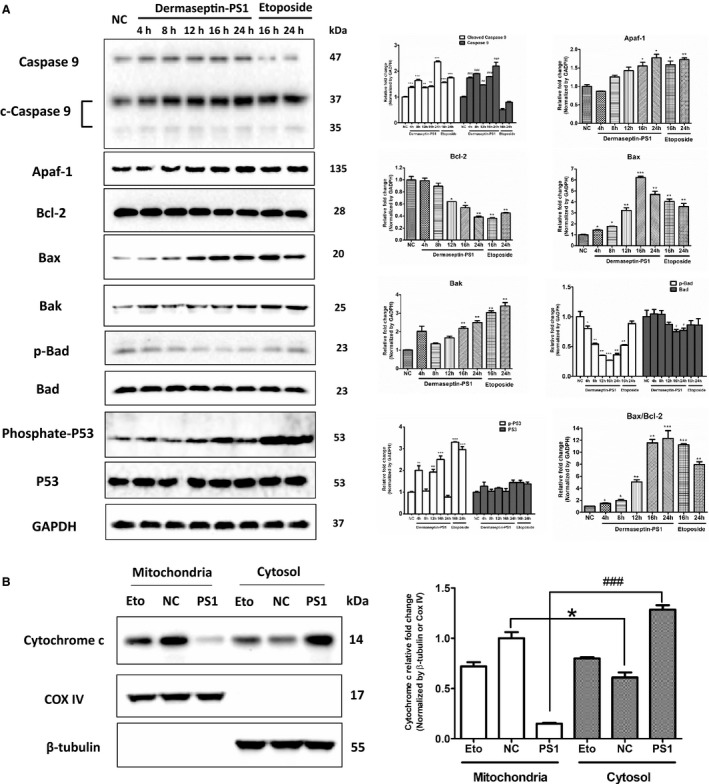
Dermaseptin‐PS1 induced U‐251 MG cell death through intrinsic apoptosis signalling. A, Protein expression of caspase 9/cleaved caspase 9, Apaf‐1, Bcl‐2, Bax, Bak, p‐Bad, Bad, p‐p53 and p53 were analysed by Western blot in U‐251 MG cells treated for 4‐24 h with 10^−6^ M Dermaseptin‐PS1 or 16‐24 h with 20 μmol/L etoposide. The detection of GAPDH protein was used as an internal control. The signal intensity was quantified by Image Lab software and GraphPad Prism 5 software was used for statistical comparison. NC, negative control. *or ^#^ *P* < 0.01, ** or ^##^ *P* < 0.05 and *** or ^###^ *P* < 0.001 vs NC. B, Cytochrome c release from the mitochondria into the cytosol was measured via Western blot. COX IV was used as a loading control for mitochondrial fractions. The signal intensity was quantified by Image Lab software and GraphPad Prism 5 software was used for statistical comparison. NC, negative control; PS1, Dermaseptin‐PS1; Eto, etoposide. *P < 0.05 vs protein expression in cytosol after etoposide treatment; ^###^ *P* < 0.001 vs protein expression in cytosol after Dermaseptin‐PS1 treatment

Then, we examined whether the intrinsic apoptotic signalling was involved. After treated with 10^−6^ M Dermaseptin‐PS1 or 20 μmol/L etoposide (Figure 6A), the expression levels of cleaved caspase 9, apoptotic protease activating factor 1 (Apaf‐1), Bcl2‐associated X protein (Bax), Bcl‐2 homologous antagonist/killer (Bak) and phosphate p53 at Ser15 were remarkedly increased. While the expression of Bcl‐2‐associated death promoter (Bad) remained stable, but the phosphorylation form of Bad, phosphate Bad at Ser112, was significantly decreased after 16 hours treatment of Dermaseptin‐PS1. Moreover, the protein expression of Bcl‐2 showed the same trend in the mRNA alteration.

Following on from this, we directly detected the cytochrome c levels in both cytoplasm and mitochondria, as shown in Figure 6B, after Dermaseptin‐PS1 treatment for 16 hours, the cytosolic cytochrome c level markedly elevated compared with the vehicle control, which suggested the mitochondrial‐related cytochrome c release, reflecting that the intrinsic apoptotic pathway had been activated.

## 4 DISCUSSION

Although, in the past decades, significant progress has been made for the treatment of cancers via induction of apoptosis, many challenges still remain, which are not only in the field of expanding the application of targeting apoptosis in multiple cancer cells, but also preventing cancer cells resistant to therapies.^12^ A novel type of drugs, named “BH‐3 mimetics,” has been developed and applied clinically, they directly inhibit Bcl‐2 family members for apoptosis activation, with the aim of overcoming the upstream proteins initiated therapeutic resistance.^29^ However, these intrinsic apoptotic pathway targeted drugs also face the problems that toxicities need to be improved in the meantime of keeping or enhancing the potency on killing cancer cells.^30^, ^31^, ^32^ In this study, we focused on investigating the potential anticancer mechanism of the novel characterised peptide Dermaseptin‐PS1 on malignant glioblastoma U‐251 MG cells.

Our data confirmed that Dermaseptin‐PS1 possessed strong antimicrobial (*E. coli*,* S. aureus* and *C. albicans*), anticancer (U‐251 MG) and haemolytic activities at the concentration of 10^−5^ M, through the morphological (Figure 3B) and LDH (Figure 3C) analysis, we presumed that these effects were resulted from necrosis caused by Dermaseptin‐PS1, which is a non‐specific cell membrane disruption mechanism, and this could be further unambiguously determined by the most reliable transmission electron microscopic detection.^33^ While the U‐251 MG cell viability decrease after being exposed to 10^−6 ^M peptide attracted our attention, as all the results implied that no mechanic cell membrane damage was likely to be involved. As a consequence, we examined the apoptosis activation. One of the interesting issues we observed was that, almost all mRNA levels of the measured apoptotic related genes (intrinsic and extrinsic) increased significantly after treatment of 10^−6^ M Dermaseptin‐PS1, but we barely seen the up‐regulation of protein level of cleaved caspase 8, the main executor of extrinsic pathway. As the fact that caspases exist in the form of zymogens, their activities depend largely on cleaving the zymogens to the active forms,^34^ and thus, compared with transcriptional‐regulation, post‐translational regulations are more essential for the caspases activity. In addition, the expression of FADD had no significant changes. Thus, we deduced that the extrinsic pathway is not involved in the Dermaseptin‐PS1 induced apoptosis. Nevertheless, the detailed mechanism associated with why the up‐regulated mRNA level of extrinsic targets has not contributed to the active form of caspase 8 warrants further investigations.

In addition, the marker proteins from the intrinsic apoptotic cascade (Figure 6A) indicated that the intrinsic apoptotic cascade was responsible for low concentration of Dermaseptin‐PS1 induced cell death. Although the cleaved caspase 9 levels were increased after exposure to Dermaseptin‐PS1, the caspase 9 level was also increased compared with the positive control etoposide treatment. We suspect that, apart from activation of caspase 9, Dermaseptin‐PS1 could also increase the expression of this gene, which culminates in elevation of both caspase 9 and its cleaved form.

Overall, our study not only provides new candidate for the treatment of malignant glioblastoma, but also illuminates insights into the preliminary mechanism by which Dermaseptin‐PS1 induces U‐251 MG cells intrinsic apoptosis at the specific 10^−6^M concentration. Therefore, we emphasise that precisely control the dose of the prescription in clinic might be another perspective research area for achieving our ultimate goal of cancer treatment.

## CONFLICT OF INTEREST

The authors declare that the research was conducted in the absence of any commercial or financial relationships that could be construed as a potential conflict of interest.

## AUTHOR CONTRIBUTION

Yuxin Wu and Tianbao Chen conceived and designed the experiments. Qilin Long, Lei Li, Hao Wang and Miaoran Li performed the experiments. Qilin Long, Lei Li, and Yuxin Wu analysed the data. Tianbao Chen, Mei Zhou, and Lei Wang contributed reagents/materials/analysis tools. Qilin Long and Yuxin Wu wrote the paper. Yuxin Wu, Lei Li and Qiaozhu Su edited the paper.
